# Americans favor predistributive over redistributive economic policies

**DOI:** 10.1073/pnas.2617191123

**Published:** 2026-08-10

**Authors:** David Broska, Jonne Kamphorst, Robb Willer

**Affiliations:** ^a^ Department of Sociology, Stanford University, Stanford, CA 94305; ^b^ Center for Socio- Political Data and Center for European Studies and Comparative Politics, Sciences Po, 75007 Paris, France

**Keywords:** predistribution, redistribution, economic inequality, public policy, public opinion

## Abstract

Redistributive policies designed to reduce economic inequality through taxes and transfers (e.g., safety-net benefits funded by progressive taxation) receive less support in the United States than in many Western democracies. Recent work suggests Americans may be more receptive to “predistributive” policies, which seek to reduce economic inequality before taxes and transfers (e.g., minimum-wage increases). Yet existing evidence leaves open whether this support gap characterizes contemporary Americans overall, and whether it persists when the same policy proposals are described in predistributive vs. redistributive terms, with their core provisions held constant. Across 31 national U.S. survey waves from 2015 to 2024 (total N=384,248), we find greater support for policies classified as predistributive than redistributive. We then fielded a preregistered experiment with a national U.S. sample (N=1,009), randomly varying whether otherwise identical policy proposals were described as increasing earnings for people lower in the income distribution, shifting resources from higher- to lower-income individuals, or presented only with the policy description given in all conditions. Support was higher under predistributive than redistributive descriptions. Comparison with the control condition clarifies this difference: Predistributive descriptions received support comparable to control, whereas redistributive descriptions received lower support. Exploratory analyses suggest perceived respect for hard work and beneficiary deservingness were associated with the support gap. Together, these results suggest Americans prefer policies that reduce inequality by influencing earnings more than through taxes and transfers.

Economic inequality (1) and persistent poverty (2) remain high in the United States, and Americans express strong preferences for reducing inequality (3, 4) and expanding economic opportunity (5). Nonetheless, compared with other Western democracies, Americans express relatively low support for welfare and other redistributive policies (6). This presents a puzzle: While Americans generally desire less economic inequality, they do not show corresponding support for the policies most commonly proposed for reducing it.

Recently, scholars have contrasted “predistribution” with redistribution. Predistributive policies are designed to reduce inequality before taxes and transfers by increasing earnings, employer-provided benefits, or bargaining power, while redistributive policies are designed to reduce inequality after market outcomes through taxes and transfers. Research in political economy finds that predistributive policies draw more support from less-educated than from more-educated voters, and links partisan realignment by education in the United States to Democratic elites’ movement away from predistribution (7). We propose that both a policy’s type and how it is described shape how predistributive or redistributive it is perceived to be, and that perception, in turn, shapes support. We ask whether contemporary Americans as a whole support predistributive over redistributive policies, and experimentally test whether describing a policy in either type’s terms shifts support. Study 1 compares support for policies whose survey descriptions present features associated with one type or the other. Study 2 holds policy type constant while varying whether descriptions emphasize raising earnings for lower-income people or shifting resources from higher- to lower-income people.

Prior research on views of inequality-reducing policies suggests several reasons why predistributive policies may avoid negative reactions Americans often have to redistributive policies. In particular, compared to redistributive policies, predistributive policies may link rewards more directly to a person’s labor (e.g., higher pay for work), aligning with evidence that economic outcomes are seen as fairer and more deserved when earned through effort (8). Further, in the United States, the inequality-reducing effects of redistribution are typically divided into separate policies, progressive taxation and transfers to the needy, with citizens rarely seeing the full cycle of who pays and who benefits (9). Predistributive policies combine who pays and who benefits in a single policy, such as a minimum wage paid by employers. Additionally, these policies, by increasing lower-income individuals’ earnings, may be seen as increasing respect paid to hard-working people, whereas redistribution may be seen as conveying rewards regardless of recipients’ efforts and deservingness (8, 9).

In Study 1, we first identified questions measuring support for predistributive or redistributive policies in 31 probability and nonprobability U.S. sample surveys fielded between 2015 and 2024. In Study 2 we fielded a preregistered experiment with a national U.S. quota-matched sample (N=1,009), randomizing whether each of six otherwise identical policy proposals was described as increasing earnings for people lower in the income distribution (predistribution), shifting resources from higher- to lower-income individuals (redistribution), or presented only with the brief policy description given in all conditions (control). For each policy, participants reported policy support, support for a candidate who promoted the policy, and perceptions of each policy.

## Results

### Study 1.

**Fig. 1.**
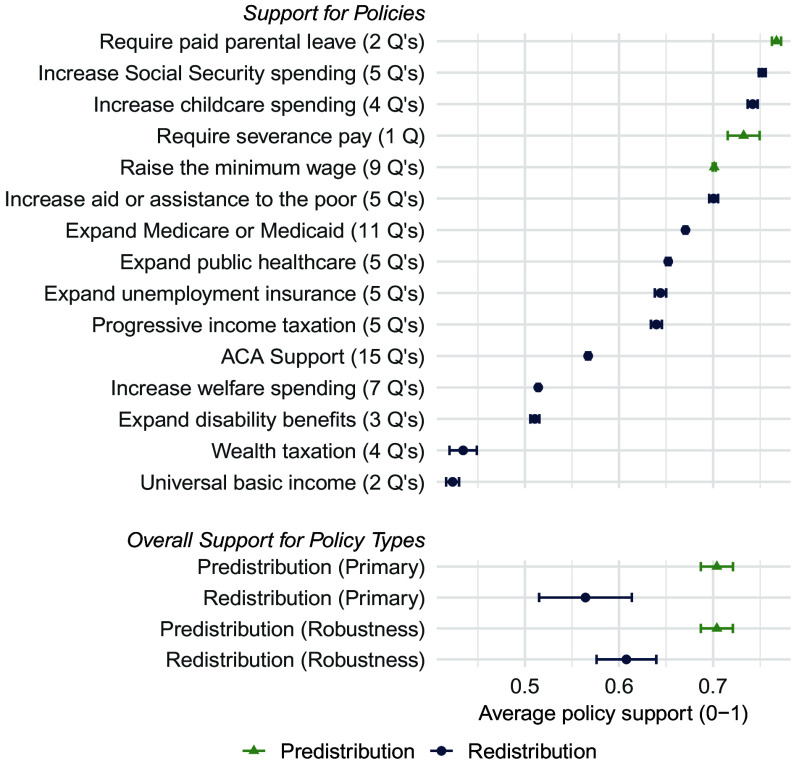
Average support for groups of substantively similar policy support questions and predistributive and redistributive policies overall, rescaled to 0–1 and reported with 95% CIs. Number of question fieldings combined into each estimate given in parentheses; the same question asked in different survey waves is counted separately. Primary analyses include clear predistributive and redistributive questions (*SI Appendix*); robustness analyses add healthcare policy support questions.

Americans reported stronger support for predistributive than redistributive policies in national U.S. survey waves (b=0.14, 95% CI = [0.09, 0.19], P<0.001; Fig. 1), equivalent to 14 points on a 0 to 100 support scale, or 0.34 SD. Support varied across policy groupings, especially among redistributive policies (Fig. 1). The gap attenuated but remained statistically significant in a robustness analysis that added healthcare questions to the redistributive category (b=0.10, 95% CI = [0.06, 0.13], P<0.001).

### Study 2.

**Fig. 2.**
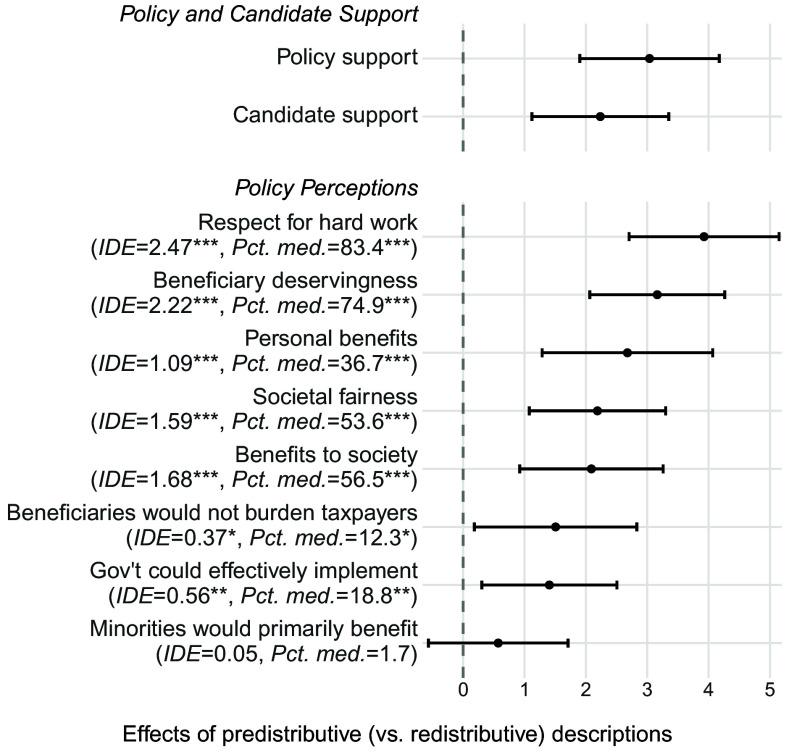
Estimated effects of predistributive (vs. redistributive) descriptions on policy and candidate support (*Top*) and policy perceptions (*Bottom*) with 95% CIs. Statistics in parentheses are noncausal indirect-effect estimates from models entering each perception separately as a mediator of policy support, followed by estimated percentage of total effect mediated (***P<0.001, **P<0.01, and *P<0.05).

When the same policies were described in predistributive rather than redistributive terms, participants expressed greater policy support (b=3.04, 95% CI = [1.90, 4.17], P<0.001; approximately 3 points on the 0 to 100 scale, or 0.11 SD) and candidate support (b=2.24, 95% CI = [1.12, 3.35], P<0.001; Fig. 2). Given partisan differences over economic policy, we explored possible heterogeneity by partisanship, finding that the policy-support difference was significantly larger among Republicans (b=4.86, 95% CI = [3.10, 6.63], P<0.001) than among Democrats (b=1.16, 95% CI = [−0.50, 2.83], P=0.171), with Independents falling between these groups (b=3.94, 95% CI = [0.72, 7.17], P=0.017). In the full sample, comparisons to the control condition clarified the overall pattern: Policy support did not differ between predistributive and control descriptions (b=0.26, 95% CI = [−0.87, 1.40], P=0.649), whereas redistributive descriptions received lower support than control descriptions (b=−2.77, 95% CI = [−3.91, −1.64], P<0.001). Thus, the predistribution-redistribution difference reflected lower support for redistributive descriptions, not higher support for predistributive descriptions. Full sample mean support remained high in all conditions: $M_{\mathrm{Predistributive}}=72.0$, $M_{\mathrm{Control}}=71.6$, and $M_{\mathrm{Redistributive}}=68.9$.

As a manipulation check, participants rated how predistributive vs. redistributive they perceived each policy to be (*SI Appendix*, section A.11). Relative to the redistributive description, the predistributive description raised perceptions of the policy as predistributive rather than redistributive (b=9.07 on the 0 to 100 composite, 95% CI = [7.61, 10.53], P<0.001). The effect of descriptions on support varied across policies (treatment-by-policy interaction, P<0.001): The descriptions shifted perceived type for all six policies but most for the EITC, Medicare For All Who Want It, and free community college, and these were the only three for which redistributive descriptions lowered support; predistributive descriptions did not raise support for any policy. With six policies, however, we cannot separate perceived type from other policy features, so we treat these differences as suggestive (per-policy estimates are on OSF).

Predistributive descriptions led participants to see the same policies as more respectful of hard work, more beneficial to deserving recipients, fairer, more beneficial to themselves, to society, and more effectively implementable by government, while reducing perceptions that beneficiaries burden taxpayers (Fig. 2). Exploratory single-mediator models analyzing posttreatment perceptions suggest that perceived respect for hard work and beneficiary deservingness were most strongly associated with the policy-support difference.

## Discussion

Americans favor predistributive over redistributive policies in recent national surveys. In the experiment, this support gap was not merely a byproduct of comparing different policies: When policy proposals’ material features were held constant, redistributive descriptions received lower support than both predistributive and control descriptions. The support difference is driven by aversion to redistribution rather than enthusiasm for predistribution: A redistributive description lowered support, while a predistributive one did not raise it. These results are limited to the contemporary United States, where deservingness norms are especially salient (8). Whether the penalty for redistributive descriptions weakens where solidarity norms are stronger is a promising question for future comparative work. Predistributive and redistributive descriptions also differ in valence, and future work could vary a gain-vs.-loss contrast directly; in our data, however, the support difference was associated more with deservingness and respect perceptions than with the perception that beneficiaries burden taxpayers. Our claims concern public support for policies as they are described and understood, not their actual economic efficiency or distributional effects.

Our findings carry implications for both policy design and policy communication. For design, policies that reduce inequality by raising earnings before taxes and transfers draw broader support than those that work through taxes and transfers, and so may be more politically viable. For communication, description moved support for the EITC, Medicare For All Who Want It, and free community college, the policies whose perceived type the descriptions also shifted most, but not for clearly typed policies like the minimum wage. Advocates thus likely have more room to shape support through framing where a policy’s type is open to more than one reading than for well-understood policies, whose support is governed by the actual policy features.

## Materials and Methods

### Study 1.

We created a dataset of policy-support questions from publicly available U.S. national survey waves fielded between 2015 and 2024. We retained questions meeting three inclusion criteria: i) the question concerns an actual or proposed government policy, ii) it measures public support for that policy on a scale interpretable as more or less support, and iii) the policy is designed to directly or indirectly reduce inequality in the distribution of wealth or income among Americans. Our search for such questions proceeded in two stages. In the first stage, we searched major U.S. national probability-sample surveys with broad policy batteries: the American National Election Studies (ANES), the General Social Survey (GSS), and the Gallup Poll Social Series (Health and Healthcare; Economy and Personal Finance). In the second stage, we extended the search to additional national surveys; this added questions from the Understanding America Study (UAS), the Rutgers Heldrich Center Work Trends surveys, and the Cooperative Election Study (CES), which uses a nonprobability sampling design. The first author and a research coordinator then independently classified each retained question on three dimensions, its fundamental type, the policy features its wording makes salient, and whether the wording stated the policy’s inequality-reduction logic (full criteria, item-level codes, and intercoder reliability in *SI Appendix*, sections A.2 and A.3). The analysis uses fundamental type; the inequality-reduction logic was stated in only one of 52 items, so Study 1 compares predistributive and redistributive policies as they are ordinarily worded. Healthcare questions were retained only for the robustness analysis because they reduce inequality through in-kind insurance rather than cash transfers, so redistributive effects depend on uptake and financing the wording does not specify (*SI Appendix*, section A.4). The resulting dataset comprises 31 national survey waves with 83 question fieldings of 52 distinct questions (question wording in *SI Appendix*, section A.5), producing 1,204,334 responses from 384,248 distinct respondents. To estimate the overall gap in support between predistributive and redistributive policies, we fit an OLS model with a binary indicator for questions about predistributive (vs. redistributive) policies. We report two-way clustered SEs at the level of question fieldings and respondents. To estimate average support for each policy grouping (sets of survey questions addressing the same underlying policy) we fit an OLS model with policy-grouping fixed effects and SEs clustered at the respondent level. *SI Appendix*, sections A.1–A.4 detail the dataset construction and analysis.

### Study 2.

We recruited participants via CloudResearch Connect to take part in a within-subject experiment, using preregistered quotas representative of the US population across gender, race, education, and party ID. After applying our preregistered exclusion criteria, our final sample comprised 5,971 responses from 1,009 participants. These participants read descriptions of six policies in random order and answered questions after each policy: the Earned Income Tax Credit, increasing the federal minimum wage, progressive income taxation, the Protecting the Right to Organize Act, Medicare For All Who Want It, and America’s College Promise Act. We selected these policies because they could be credibly described through features associated with both policy types. All three conditions presented the same baseline description of the policy, thus controlling for policy type, while varying the described inequality-reduction logic (predistributive, redistributive, or, in the control condition, unstated). The control condition showed only the baseline description, while the other conditions appended additional, short text. The predistributive text emphasized how the policy increases earnings for lower-income individuals; the redistributive text emphasized how it shifts resources from higher-income individuals, employers, or owners to lower-income people or workers. Each experimental condition—control, predistribution, and redistribution—appeared twice per participant. We estimate treatment effects using linear mixed-effects models with random intercepts at the participant level as well as a set of pretreatment control variables (*SI Appendix*, section A.7). See *SI Appendix*, sections A.9 and A.11 for wording of all variables and *SI Appendix*, section A.10 for full policy descriptions used in each condition. Research was approved by the Stanford University Institutional Review Board. All subjects provided informed consent.

## Supplementary Material

Appendix 01 (PDF)

## Data Availability

Preregistration, data needed to reproduce the analyses, materials, and code are available on OSF: https://osf.io/kndq7 (10).
