# Supplementary material for: Americans favor predistributive over redistributive economic policies
Source: Proc Natl Acad Sci U S A. 2026 Aug 10;123(33):e2617191123. doi: 10.1073/pnas.2617191123 (PMC13486588; doi:10.1073/pnas.2617191123)
Supplement: Supplementary file 1 [file pnas.2617191123.sapp.pdf]

Supporting Information for

Americans favor predistributive over redistributive economic policies

David Broska[1,2,3], Jonne Kamphorst[2,4], Robb Willer[1,3]

[1] Corresponding authors

[2] Authors contributed equally and are listed alphabetically

[3] Department of Sociology, Stanford University, Stanford, CA 94305

[4] Center for Socio-Political Data and Center for European Studies and Comparative Politics, Sciences Po, 75007 Paris, France

Corresponding authors:

David Broska

Department of Sociology, Stanford University, Stanford, CA 94305

dbroska@stanford.edu

Robb Willer

Department of Sociology, Stanford University, Stanford, CA 94305

willer@stanford.edu

This PDF file includes:

Supplementary Methods and Materials

SI References

A. Supporting Information

A.1 Data Collection (Study 1)

Search for survey datasets. Our search proceeded in two stages. In the first stage, we searched major U.S. national probability-sample surveys with broad policy batteries fielded between 2015 and 2024: the American National Election Studies (ANES), the General Social Survey (GSS), and the Gallup Poll Social Series (Health and Healthcare; Economy and Personal Finance). For each survey wave, research assistants reviewed the full questionnaire and identified every economic-policy-support question, which we define as a question asking the respondent whether they support or oppose an actual or proposed government economic policy. In the second stage, we extended the search to additional national surveys, including those based on non-probability sampling designs as well as probability-based surveys with narrower or topic-specific policy content. The second-stage search added questions from the Cooperative Election Study (CES), the Understanding America Study (UAS), and the Rutgers Heldrich Center Work Trends surveys. We also reviewed additional U.S. national surveys that contained no questions meeting our inclusion criteria, including the Pew Research Center's American Trends Panel and AP-NORC Center for Public Affairs Research polls.

Inclusion criteria. We retained survey questions that satisfied three criteria. First, the question concerns an actual or proposed government policy that is sufficiently specific to be classifiable as predistributive or redistributive. Please refer to Section A.5 for the list of included questions. Our first criterion excluded general statements about government action without reference to a specific policy (e.g., "the government should take measures to reduce differences in income levels"), responsibility allocation (e.g., "Is it the responsibility of the federal government to make sure all Americans have healthcare coverage?"), and questions that bundle multiple policies (e.g., "Do you support each of the following proposals? Spend $2.2 trillion over the next decade to provide universal prekindergarten, subsidies for child care, expanded financial aid for college, housing support, home and community care for older Americans, and to shift the U.S. economy away from fossil fuels to renewable energy and electric cars.").

Second, the question measures public support for that policy on a scale interpretable as more or less support. This criterion excluded items asking about a policy's perceived effects rather than support for the policy (e.g., whether "the No Child Left Behind Act made U.S. education better or worse"), and items measuring general attitudes or feelings toward an actor or institution rather than support for a specific policy (e.g., feeling-thermometer questions asking respondents to "rate how you feel toward labor unions on a scale from 0 to 100"). Some questions asked whether the government spends "too much," "too little," or "about the right amount" on a program. We treated "too little" as the highest-support response.

Third, the policy is designed to directly or indirectly reduce inequality in the distribution of wealth or income among Americans. By "designed to," we mean that a primary intended function of the policy appears to be reducing economic inequality. We focus on the apparent intended purpose of a policy rather than assessments of the likelihood and extent to which the policy will, in fact, reduce inequality, both because the latter is difficult to

determine and because policy support is more likely to be shaped by apparent intended functions than by impact evaluations.

Final dataset. The resulting dataset comprises 31 national survey waves from the American National Election Studies (ANES), the Cooperative Election Study (CES), the Gallup Poll Social Series (Health and Healthcare; Economy and Personal Finance), the General Social Survey (GSS), the Understanding America Study (UAS), and the Rutgers Heldrich Center Work Trends surveys (the 2020 pre- and post-election waves and the April 2023 wave) (3-17). The final dataset includes 83 question fieldings. These 83 fieldings correspond to 52 distinct questions. When the same question is fielded in multiple survey waves, we count each fielding separately. For example, the paid parental leave question appears in ANES 2016 and ANES 2020, yielding two question fieldings in the dataset. Figure 1's parenthetical counts in the main text use this convention. Section A.5 reports the wording of each question and the survey waves in which it appeared. Within these waves, 384,248 distinct respondents answered the questions fielded in their wave, producing 1,204,334 responses in total.

A.2 Coding procedure

The first author and a research coordinator independently classified all unique policy descriptions from Studies 1 and 2 on three dimensions (A, B, and C). Disagreements were resolved through discussion. Procedures for coding the three dimensions and possible classifications are given below in the same form they were given to coders. Coders were given the following instructions:

Code each item independently, WITHOUT looking at anyone else's codes. Give ratings in all the three columns: A_FundamentalType, B_SalientFeatures, and C_DescribedLogic, using only the values listed below. The three columns are dimensions A, B, and C defined here.

A. *Fundamental type.* The policy's actual character, independent of how it is described. Values:

- Predistributive: policy is designed to reduce inequality before taxes and transfers, by raising earnings, wages, and/or employer-provided benefits.
- Redistributive: policy is designed to reduce inequality after market outcomes, through taxes and government transfers or benefits.
- Conditionally redistributive: policy is designed to reduce inequality after market outcomes, but works through in-kind insurance against risk rather than through cash transfers or changes in earnings, so the degree of redistribution turns on use of the covered benefit and on financing the policy does not specify. Note: health-insurance and health-care access policies (for example, expanding access to Medicare or Medicaid) will typically be coded conditionally redistributive. A policy that delivers a defined cash benefit or targeted cash assistance is coded redistributive even when its financing is left unspecified.
- Both: policy includes both predistributive and redistributive structural features.
- Neither: policy is not designed to reduce economic inequality at all, or it is not designed to do so in a way classifiable as "predistributive" or "redistributive" according to the above coding rules.

B. *Salient features.* If a policy's structural features are named in policy description, what fundamental type (listed above in A) do they correspond to? Values:

- Predistributive,
- Redistributive,
- Conditionally redistributive,
- Both (both predistributive and redistributive structural features of the policy are named in policy description), or,
- Neither (structural features are not named, or the structural features that are named do not correspond to a policy classifiable as "predistributive" or "redistributive").

Note: a policy that is only named and its structural features are not described should be classified as "neither."
Note: Values for B will typically align with values for A, with the most common exceptions likely to be when the policy's features are not described at all.
Note: criterion B refers to the structural features of the policy appearing in the description, not the inequality-reduction logic that may appear in the description (which is captured by C, see below).

C. *Described logic.* Does the policy description state how the policy is designed to reduce inequality? If yes, which policy type does the described way of reducing inequality correspond to? Values:

- Predistributive: policy description presents the policy as reducing inequality by raising earnings, wages, and/or employer-provided benefits,
- Redistributive: policy description presents the policy as reducing inequality through taxes and government transfers or benefits,
- Both: policy description presents the policy as reducing inequality in both predistributive and redistributive ways
- Neither: There is no policy description, or no policy description that describes how the policy is designed to reduce inequality, or any presented policy description does not describe the policy as reducing inequality in a way classifiable as "predistributive" or "redistributive" according to above coding rules.

A.3 Coding of Policy Types and Policy Descriptions

The two coders' codes agreed perfectly on two of the three dimensions. Across both studies, agreement was 100% for (A) fundamental type ($\kappa = 1.00$) and for (C) described logic ($\kappa = 1.00$). For salient features – the most interpretive dimension, capturing whether a description names a policy's features or merely gives the policy's name – agreement was 73% across both studies ($\kappa = 0.64$). All disagreements were resolved through discussion. Final codes for all policy descriptions, following reconciliation, are listed below. For each dimension, we list the final codes given to each Study 1 question and each Study 2 policy x description combination. The survey questions corresponding to the Study 1 codes are given below in A.5. The unique policy x description combinations corresponding to the Study 2 codes are given below in A.10.

Study 1

- *A. Fundamental Type (Study 1):* Predistributive: Q1, Q6–Q13. Conditionally redistributive: Q16–Q27, Q36–Q40. Redistributive: Q2–Q5, Q14–Q15, Q28–Q35, Q41–Q52. (None: both, neither.)
- *B. Salient Features (Study 1):* Predistributive: Q1, Q6–Q13. Conditionally redistributive: Q17–Q21, Q23–Q24, Q40. Redistributive: Q2–Q5, Q14–Q15, Q28, Q30–Q35, Q41–Q52. Neither: Q16, Q22, Q25–Q27, Q29, Q36–Q39. (None: both.)
- *C. Described Logic (Study 1):* Redistributive: Q48. Neither: Q1–Q47, Q49–Q52. (None: predistributive, both.)

Study 2

- *A. Fundamental Type (Study 2)*: Predistributive: P2p, P2r, P2c, P4p, P4r, P4c. Redistributive: P1p, P1r, P1c, P3p, P3r, P3c. Conditionally redistributive: P5p, P5r, P5c. Both: P6p, P6r, P6c. (None: neither)
- *B. Salient Features (Study 2)*: Predistributive: P2p, P2r, P2c, P4p, P4r, P4c. Redistributive: P3p, P3r, P3c. Conditionally redistributive: P5p, P5r, P5c. Both: P1p, P1r, P1c, P6p, P6r, P6c. (None: neither)
- *C. Described Logic (Study 2)*: Predistributive: P1p, P2p, P3p, P4p, P5p, P6p (all six predistributive descriptions). Redistributive: P1r, P2r, P3r, P4r, P5r, P6r. Neither: P1c, P2c, P3c, P4c, P5c, P6c (the six control descriptions). (None: both)

A.4 Data analysis (Study 1)

We estimate the following two OLS regressions on our pooled survey dataset with observations varying across survey programs (s), years (t), respondents (i), survey questions (q), and policy groupings (g). We use $g(q)$ to denote the policy grouping to which survey question q belongs.

Model 1. Support for Policies

$$Y_{iqst} = \alpha + \lambda_{g(q)} + \epsilon_{iqst}$$

Model 2. Overall Support for Policy Types

$$Y_{iqst} = \alpha + \gamma D_q + \epsilon_{iqst}$$

The outcome Y_{iqst} refers to policy support, recoded so that higher values indicate more support and rescaled to the 0–1 interval. In Model 1, we estimate a set of fixed effects for policy groupings, denoted as $\lambda_{g(q)}$. In Model 2, D_q is an indicator variable for whether question q is classified as predistributive ($D_q = 1$) or redistributive ($D_q = 0$). The coefficient γ estimates the difference in mean support between predistributive and redistributive policies.

For Model 2, we report two-way cluster-robust standard errors. We cluster at the level of question fieldings – treating each fielding of a question in a survey wave as a separate cluster – because the key regressor is defined at this level. We cluster at the respondent level because respondents answer multiple policy questions. For Model 1, we cluster only at the respondent level. We do not also cluster at the level of question fieldings because some policy groupings are represented by only a single question fielding, making question-level clustered uncertainty not well identified for all group means.

Figure 1 reports per-group averages with 95% confidence intervals from these models. For Model 2, we report results from two specifications: a primary specification and a robustness specification. The robustness specification additionally includes three sets of healthcare policies classified as conditionally redistributive: "Expand Medicare or Medicaid," "Expand public healthcare," and "ACA Support" (see Section A.5).

While this manuscript focuses on parsimonious model specifications (Models 1 and 2), we briefly note two alternative analytical strategies we do not report here. First, we do not include additive fixed effects for survey program and year. Such fixed effects would identify the predistribution–redistribution coefficient (γ) only from survey waves in which questions about both predistributive and redistributive policies were fielded (1). In our dataset, four of seven survey programs (GSS, UAS, Gallup Health and Healthcare series, and the Gallup Economy and Personal Finance series) include only redistributive questions, and four of ten fielding years (2015, 2017, 2023, and 2024) cover only one category. Second, we do not report estimates using the survey-provided sampling weights. Neither survey weights nor fixed effects substantively change the estimated difference in support for predistributive and redistributive policies.

A.5 Survey Question Wording (Study 1)

This section documents the survey questions underlying each policy grouping in Fig. 1, in the same order as the figure. For each grouping, we report the number of question fieldings (counting each fielding across survey waves separately) and list each question's classification (predistribution, redistribution, or conditionally redistributive), wording, survey wave(s) and variable name(s), and response scale. Each question is assigned a unique label, Q1–Q52, in the order listed here; these labels index the questions in Policy Types and Policy Descriptions section in A.3.

Require paid parental leave (2 question fieldings)

1. Require employers to offer paid parental leave (Q1)

- Classification: Predistribution
- Question: "Do you favor, oppose, or neither favor nor oppose requiring employers to offer paid leave to parents of new children?"
- Survey wave: ANES 2016 (V161226x), ANES 2020 (V201405x)
- Scale: Oppose a great deal (0), Oppose a moderate amount (1), Oppose a little (2), Neither favor nor oppose (3), Favor a little (4), Favor a moderate amount (5), Favor a great deal (6)

Increase Social Security spending (5 question fieldings)

1. Increase federal spending on Social Security (Q2)

- Classification: Redistribution
- Question: "What about Social Security? Should federal spending on Social Security be increased, decreased, or kept the same?"
- Survey wave: ANES 2016 (V161205), ANES 2020 (V201302x)
- Scale (ANES 2016): Decreased (0), Kept the same (1), Increased (2)
- Scale (ANES 2020): Decreased a lot (0), Decreased a little (1), Kept the same (2), Increased a little (3), Increased a lot (4)

2. Increase spending on Social Security (Q3)

- Classification: Redistribution
- Question: "... are we spending too much, too little, or about the right amount on Social Security?"
- Survey wave: GSS 2021 (natsoc), GSS 2022 (natsoc), GSS 2024 (natsoc)
- Scale: Too much (0), About right (1), Too little (2)

Increase childcare spending (4 question fieldings)

1. Increase spending on childcare assistance (Q4)

- Classification: Redistribution
- Question: "... are we spending too much, too little, or about the right amount on Assistance for childcare?"
- Survey wave: GSS 2021 (natchld), GSS 2022 (natchld), GSS 2024 (natchld)
- Scale: Too much (0), About right (1), Too little (2)

2. Increase federal spending on child care (Q5)

- Classification: Redistribution
- Question: "What about Child care? Should federal spending on child care be increased, decreased, or kept the same?"
- Survey wave: ANES 2016 (V161210)
- Scale: Decreased (0), Kept the same (1), Increased (2)

Require severance pay (1 question fielding)

1. Importance of requiring severance pay for layoffs (Q6)

- Classification: Predistribution
- Question: "(Next, I will read you/we will show you actions that the federal government might take to help workers or those looking for better jobs. For each one, please indicate whether you think that these actions are extremely important, very important, somewhat important, not very important, or not at all important.) Is it extremely important, very important, somewhat important, not very important, or not at all important that the federal government...Require employers to offer severance payment equal to one week's pay for every year of employment to people who are going to lose their jobs?"
- Survey wave: Work Trends April 2023 (work9d)
- Scale: Not at all important (0), Not very important (1), Somewhat important (2), Very important (3), Extremely important (4)

Raise the minimum wage (9 question fieldings)

1. Raise the minimum wage (Q7)

- Classification: Predistribution
- Question: "Should the minimum wage be raised, kept the same, lowered but not eliminated, or eliminated altogether?"
- Survey wave: ANES 2016 (V162192)
- Scale: Eliminated altogether (0), Lowered but not eliminated (1), Kept the same (2), Raised (3)

2. Raise the federal minimum wage (Q8)

- Classification: Predistribution
- Question: "Should the federal minimum wage be raised, kept the same, lowered but not eliminated, or eliminated altogether?"
- Survey wave: ANES 2020 (V202377)
- Scale: Eliminated altogether (0), Lowered but not eliminated (1), Kept the same (2), Raised (3)

3. Raise the minimum wage to $15 (Q9)

- Classification: Predistribution
- Question: "Raise the minimum wage to $15 an hour."
- Survey wave: CES 2020 (CC20_350b), CES 2019 (CC19_328b)
- Scale: Oppose (0), Favor (1)

4. Increase the minimum wage paid to federal contractors to $15 an hour (Q10)

- Classification: Predistribution
- Question: "Increase the minimum wage paid to federal contractors to $15 an hour"
- Survey wave: CES 2021 (CC21_355e), CES 2022 (CC22_355d)
- Scale: Oppose (0), Support (1)

5. Raise federal minimum wage to $12 by 2020 (Q11)

- Classification: Predistribution
- Question: "Congress considers many issues. If you were in Congress would you vote FOR or AGAINST each of the following? Minimum wage. Raises the federal minimum wage to $12 an hour by 2020."
- Survey wave: CES 2016 (CC16_351K)
- Scale: Against (0), For (1)

6. Raise state minimum wage to $12 (Q12)

- Classification: Predistribution
- Question: "If your state put the following questions for a vote on the ballot, would you vote FOR or AGAINST? Raise the state minimum wage to $12 an hour."
- Survey wave: CES 2018 (CC18_414A)
- Scale: Against (0), For (1)

7. Raise federal minimum wage to at least $15 (Q13)

- Classification: Predistribution
- Question: "To what degree do you agree or disagree with the following statements? Government should raise the federal minimum wage to at least $15 per hour."
- Survey wave: Work Trends post-election December 2020 (Q22B_A)
- Scale: Strongly disagree (0), Somewhat disagree (1), Somewhat agree (2), Strongly agree (3)

Increase aid or assistance to the poor (5 question fieldings)

1. Increase federal spending on aid to the poor (Q14)

- Classification: Redistribution
- Question: "What about aid to the poor? Should federal spending on aid to the poor be increased, decreased, or kept the same?"
- Survey wave: ANES 2016 (V161211), ANES 2020 (V201320x)
- Scale (ANES 2016): Decreased (0), Kept the same (1), Increased (2)
- Scale (ANES 2020): Decreased a lot (0), Decreased a little (1), Kept the same (2), Increased a little (3), Increased a lot (4)

2. Increase spending on assistance to the poor (Q15)

- Classification: Redistribution
- Question: "... are we spending too much, too little, or about the right amount on Assistance to the poor?"
- Survey wave: GSS 2021 (natfarey), GSS 2022 (natfarey), GSS 2024 (natfarey)
- Scale: Too much (0), About right (1), Too little (2)

Expand Medicare or Medicaid (11 question fieldings)

1. Provide Medicare for all Americans (Q16)

- Classification: Conditionally redistributive
- Question: "Thinking now about health care policy, would you support or oppose each of the following proposals? Provide Medicare for all Americans."
- Survey wave: CES 2018 (CC18_327a)
- Scale: Oppose (0), Support (1)

2. Expand Medicare to cover all Americans (Q17)

- Classification: Conditionally redistributive
- Question: "Thinking now about health care policy, would you support or oppose each of the following

proposals? Expand Medicare to a single comprehensive public health care coverage program that would cover all Americans."
- Survey wave: CES 2020 (CC20_327a), CES 2021 (CC21_320a), CES 2022 (CC22_327a), CES 2019 (CC19_327a)
- Scale: Oppose (0), Support (1)

3. Expand Medicaid to cover low-income individuals/families (Q18)

- Classification: Conditionally redistributive
- Question: "Thinking now about health care policy, would you support or oppose each of the following proposals? Expand Medicaid to cover individuals making less than $25,000 and families making less than $40,000 a year."
- Survey wave: CES 2021 (CC21_320d)
- Scale: Oppose (0), Support (1)

4. Oppose major cuts to Medicare and Medicaid (Q19)

- Classification: Conditionally redistributive
- Question: "A Budget Plan that would cut Medicare and Medicaid by 40%. Would reduce debt by 16% by 2020."
- Survey wave: CES 2015 (CC15_330a)
- Scale: Support cuts (0), Oppose cuts (1)

5. Make Medicare available as a public option (Q20)

- Classification: Conditionally redistributive
- Question: "Thinking now about health care policy, would you support or oppose the following proposal? Make Medicare available as an option to anyone, but allow people to keep private insurance if they prefer."
- Survey wave: CES 2019 (CC19_327b)
- Scale: Oppose (0), Support (1)

6. Lower Medicare eligibility age from 65 to 50 (Q21)

- Classification: Conditionally redistributive
- Question: "Thinking now about health care policy, would you support or oppose the following proposal? Lower the eligibility age for Medicare from 65 to 50."
- Survey wave: CES 2019 (CC19_327c)
- Scale: Oppose (0), Support (1)

7. Support replacing private insurance with Medicare for all (Q22)

- Classification: Conditionally redistributive
- Question: "To what extent do you support or oppose eliminating private health insurance and, in its place, providing a publicly run healthcare program similar to Medicare that all Americans would be eligible for?"
- Survey wave: UAS 199 2019 (cf032)
- Scale: Strongly oppose (0), Somewhat oppose (1), Somewhat support (2), Strongly support (3)

8. Allow buy-in to a publicly run health insurance plan like Medicare (Q23)

- Classification: Conditionally redistributive
- Question: "To what extent do you support or oppose allowing any American younger than 65 to either buy into a publicly run healthcare program similar to Medicare, or keep the plan they have?"
- Survey wave: UAS 199 2019 (cf031)
- Scale: Strongly oppose (0), Somewhat oppose (1), Somewhat support (2), Strongly support (3)

Expand public healthcare (5 question fieldings)

1. Pay higher taxes to improve healthcare for all (Q24)

- Classification: Conditionally redistributive
- Question: "How willing would you be to pay higher taxes to improve the level of health care for all people in the United States? Would you be ..."
- Survey wave: GSS 2022 (hlthtax)
- Scale: Very unwilling (0), Fairly unwilling (1), Neither willing nor unwilling (2), Fairly willing (3), Very willing (4)

2. State should increase healthcare spending (Q25)

- Classification: Conditionally redistributive
- Question: "...Would you like your legislature to increase or decrease spending on the five areas below? Health care."
- Survey wave: CES 2016 (CC16_426_2)
- Scale: Greatly decrease (0), Slightly decrease (1), Maintain (2), Slightly increase (3), Greatly increase (4)

3. Support long-term expansion of government-provided healthcare (Q26)

- Classification: Conditionally redistributive
- Question: "Do you support or oppose long-term expansions of government-provided healthcare?"

- Survey wave: UAS 280 2021 (at003b), UAS 296 Covid 2020 (at003b)
- Scale: Strongly oppose (0), Somewhat oppose (1), Neither oppose nor support (2), Somewhat support (3), Strongly support (4)

4. Support short-term expansion of government-provided healthcare (Q27)

- Classification: Conditionally redistributive
- Question: "Do you support or oppose short-term expansions of government-provided healthcare to help individuals affected by the COVID-19 pandemic?"
- Survey wave: UAS 296 Covid 2020 (at003a)
- Scale: Strongly oppose (0), Somewhat oppose (1), Neither oppose nor support (2), Somewhat support (3), Strongly support (4)

Expand unemployment insurance (5 question fieldings)

1. Support short-term expansion of unemployment benefits (Q28)

- Classification: Redistribution
- Question: "Do you support or oppose short-term expansion of unemployment benefits to help individuals who have lost their jobs during the COVID-19 pandemic?"
- Survey wave: UAS 296 Covid 2020 (at001a)
- Scale: Strongly oppose (0), Somewhat oppose (1), Neither oppose nor support (2), Somewhat support (3), Strongly support (4)

2. Support long-term expansion of unemployment benefits (Q29)

- Classification: Redistribution
- Question: "Do you support or oppose long-term expansion of unemployment benefits?"
- Survey wave: UAS 280 2021 (at001b), UAS 296 Covid 2020 (at001b)
- Scale: Strongly oppose (0), Somewhat oppose (1), Neither oppose nor support (2), Somewhat support (3), Strongly support (4)

3. Extend unemployment insurance benefits for laid-off workers (Q30)

- Classification: Redistribution
- Question: "To what degree do you agree or disagree with the following statements? Government should assist laid off workers by extending their unemployment insurance benefits."
- Survey wave: Work Trends pre-election Nov 2020 (Q22_A), Work Trends post-election December 2020 (Q22_A)
- Scale: Strongly disagree (0), Somewhat disagree (1), Somewhat agree (2), Strongly agree (3)

Progressive income taxation (5 question fieldings)

1. Increase income taxes on those making over $1M/yr (Q31)

- Classification: Redistribution
- Question: "Do you favor, oppose, or neither favor nor oppose increasing income taxes on people making over one million dollars per year?"
- Survey wave: ANES 2020 (V202325)
- Scale: Oppose (0), Neither favor nor oppose (1), Favor (2)

2. High incomes should pay larger share in taxes (Q32)

- Classification: Redistribution
- Question: "Do you think people with high incomes should pay a larger share of their income in taxes than those with low incomes, the same share, or a smaller share?"
- Survey wave: GSS 2021 (taxshare)
- Scale: Much smaller share (0), Smaller share (1), The same share (2), Larger share (3), Much larger share (4)

3. Raise taxes on high incomes, extend lower income cuts (Q33)

- Classification: Redistribution
- Question: "The Middle Class Tax Cut Act – Would increase taxes on people with incomes above $200,000, and would extend Bush era tax cuts for incomes below $200,000. Would increase the budget deficit by an estimated $250 billion."
- Survey wave: CES 2015 (CC15_330c)
- Scale: Oppose (0), Support (1)

4. Support short-term increase in income taxes on high earners (Q34)

- Classification: Redistribution
- Question: "To help offset costs associated with the COVID-19 pandemic, I support short-term increases in... Income taxes on high-income earners"
- Survey wave: UAS 296 Covid 2020 (at006s1)
- Scale: Not selected (0), Selected (1) [multi-select indicator]

5. Support long-term increase in income taxes on high earners (Q35)

- Classification: Redistribution
- Question: "I support long-term increases in... Income taxes on high-income earners"

- Survey wave: UAS 296 Covid 2020 (at007s1)
- Scale: Not selected (0), Selected (1) [multi-select indicator]

ACA Support (15 question fieldings)

1. Approve of the ACA (Obamacare) (Q36)

- Classification: Conditionally redistributive
- Question: "Do you approve, disapprove, or neither approve nor disapprove of the Affordable Care Act of 2010, sometimes called Obamacare?"
- Survey wave: ANES 2020 (V202328x)
- Scale: Disapprove a great deal (0), Disapprove a moderate amount (1), Disapprove a little (2), Neither approve nor disapprove (3), Approve a little (4), Approve a moderate amount (5), Approve a great deal (6)

2. Oppose repeal of the Affordable Care Act (Q37)

- Classification: Conditionally redistributive
- Question: "Thinking now about health care policy, would you support or oppose each of the following proposals? Repeal the entire Affordable Care Act."
- Survey wave: CES 2019 (CC19_327d), CES 2020 (CC20_327d), CES 2021 (CC21_320b), CES 2022 (CC22_327c)
- Scale: Support repeal (0), Oppose repeal (1)

3. Oppose repeal of the Affordable Care Act (2017 bills battery) (Q38)

- Classification: Conditionally redistributive
- Question: "Congress considered many important bills over the past few years. For each of the following tell us whether you support or oppose the legislation in principle. Repeal Affordable Care Act. Would repeal the Affordable Care Act."
- Survey wave: CES 2017 (CC17_340A)
- Scale: Support repeal (0), Oppose repeal (1)

4. Approve of the Affordable Care Act (Q39)

- Classification: Conditionally redistributive
- Question: "As you may know, a health care law was passed in 2010. Given what you know about the health care law, do you generally approve or disapprove of it?"
- Survey wave: GallupHealth 2015 (aca_approve), GallupHealth 2016 (aca_approve), GallupHealth 2017 (aca_approve), GallupHealth 2018 (aca_approve), GallupHealth 2019 (aca_approve),

GallupHealth 2020 (aca_approve), GallupHealth 2021 (aca_approve), GallupHealth 2024 (aca_approve)
- Scale: Disapprove (0), Approve (1)

5. Support maintaining ACA coverage (Q40)

- Classification: Conditionally redistributive
- Question: "Congress considered many important bills over the past few years. For each of the following tell us whether you support or oppose the legislation in principle. American Health Care Act. Would repeal the tax penalties on individuals for not maintaining health coverage and on employers for not offering coverage. Would end subsidies to help people purchase insurance and would end funding for states that expanded Medicaid."
- Survey wave: CES 2017 (CC17_340C)
- Scale: Support repeal (0), Oppose repeal (1)

Increase welfare spending (7 question fieldings)

1. Increase federal spending on welfare programs (Q41)

- Classification: Redistribution
- Question: "What about welfare programs? Should federal spending on welfare programs be increased, decreased, or kept the same?"
- Survey wave: ANES 2020 (V201314x)
- Scale: Decreased a lot (0), Decreased a little (1), Kept the same (2), Increased a little (3), Increased a lot (4)

2. State should increase welfare spending (2016 wording) (Q42)

- Classification: Redistribution
- Question: "State legislatures must make choices when making spending decisions on important state programs. Would you like your legislature to increase or decrease spending on the five areas below? Welfare"
- Survey wave: CES 2016 (CC16_426_1)
- Scale: Greatly decrease (0), Slightly decrease (1), Maintain (2), Slightly increase (3), Greatly increase (4)

3. State should increase welfare spending (2020 and 2022 wording) (Q43)

- Classification: Redistribution

- Question: "State legislatures must make choices when making spending decisions on important state programs. How would you like your legislature to spend money on each of the five areas below? Welfare"
- Survey wave: CES 2020 (CC20_443_1), CES 2022 (CC22_443_1)
- Scale: Greatly decrease (0), Slightly decrease (1), Maintain (2), Slightly increase (3), Greatly increase (4)

4. Increase spending on welfare (Q44)

- Classification: Redistribution
- Question: "... are we spending too much, too little, or about the right amount on Welfare?"
- Survey wave: GSS 2021 (natfare), GSS 2022 (natfare), GSS 2024 (natfare)
- Scale: Too much (0), About right (1), Too little (2)

Expand disability benefits (3 question fieldings)

1. Increase Social Security Disability benefits (Q45)

- Classification: Redistribution
- Question: "Do you agree with the following statements? The amount that disabled workers get from Social Security should be increased (even if that means raising taxes)."
- Survey wave: UAS 322 2021 (val1a)
- Scale: Strongly disagree (0), Disagree (1), Neither disagree nor agree (2), Agree (3), Strongly agree (4)

2. Reduce Social Security Disability work requirements (Q46)

- Classification: Redistribution
- Question: "Do you agree with the following statements? The work requirements for Social Security disability insurance should be reduced (even if that means raising the taxes)."
- Survey wave: UAS 322 2021 (val1c)
- Scale: Strongly disagree (0), Disagree (1), Neither disagree nor agree (2), Agree (3), Strongly agree (4)

3. Increase Social Security Disability benefits for low-income households (Q47)

- Classification: Redistribution
- Question: "Do you agree with the following statements? The amount that disabled individuals living in low-income households receive from Social Security should be increased (even if that means raising taxes)."
- Survey wave: UAS 322 2021 (val1d)
- Scale: Strongly disagree (0), Disagree (1), Neither disagree nor agree (2), Agree (3), Strongly agree (4)

Wealth taxation (4 question fieldings)

1. Government should redistribute wealth by heavy taxes on the rich (Q48)

- Classification: Redistribution
- Question: "Do you think our government should or should not redistribute wealth by heavy taxes on the rich?"
- Survey wave: GallupEconFin 2015 (govt_redistrib), GallupEconFin 2016 (govt_redistrib)
- Scale: No, should not (0), Yes, should (1)

2. Support short-term increase in wealth taxes (Q49)

- Classification: Redistribution
- Question: "To help offset costs associated with the COVID-19 pandemic, I support short-term increases in... Wealth taxes"
- Survey wave: UAS 296 Covid 2020 (at006s6)
- Scale: Not selected (0), Selected (1) [multi-select indicator]

3. Support long-term increase in wealth taxes (Q50)

- Classification: Redistribution
- Question: "I support long-term increases in... Wealth taxes"
- Survey wave: UAS 296 Covid 2020 (at007s6)
- Scale: Not selected (0), Selected (1) [multi-select indicator]

Universal basic income (2 question fieldings)

1. Support $12K/yr federal program (funded by higher taxes) (Q51)

- Classification: Redistribution
- Question: "Do you favor, oppose, or neither favor nor oppose establishing a federal program that gives all citizens $12,000 per year, provided they meet certain conditions? This program would be paid for with higher taxes."
- Survey wave: ANES 2020 (V202376x)
- Scale: Oppose a great deal (0), Oppose a moderate amount (1), Oppose a little (2), Neither favor nor oppose (3), Favor a little (4), Favor a moderate amount (5), Favor a great deal (6)

2. Support universal basic income (Q52)

- Classification: Redistribution

- Question: "Some business executives have proposed that the U.S. government should provide every American with a Universal Basic Income, that is a minimum amount of money each month so they can afford a basic level of shelter, healthcare and food. Do you support or oppose this idea?"
- Survey wave: UAS 167 2019 (ef_001)
- Scale: Oppose (0), No opinion (1), Support (2)

A.6 Data collection (Study 2)

Study 2 recruited participants through CloudResearch Connect in February 2025, using preregistered quotas representative of the U.S. population across gender, race, education, and party ID. The preregistered exclusion criteria removed duplicate responses (keeping only the first from each IP address), participants under 18, and participants who failed either of two attention checks. After applying these criteria, our final sample comprised 5,971 responses from 1,009 participants. Quota matching was assessed after applying these exclusion criteria, as preregistered.

Participants first answered pre-treatment questions (Section A.9). They then read descriptions of six policies in random order: the Earned Income Tax Credit (EITC), increasing the federal minimum wage to $12, progressive income taxation, the Protecting the Right to Organize Act, Medicare For All Who Want It, and America's College Promise Act. Each policy was randomly assigned to one of three experimental conditions – predistribution, redistribution, or control – and each condition appeared twice per participant (Section A.10). Post-treatment questions followed each policy (Section A.11).

A.7 Data analysis (Study 2)

We estimate the following preregistered model: $Y_{iq} = \beta_0 + \mathbf{D}_{iq}\boldsymbol{\beta}_D + \mathbf{Policy}_q\,\boldsymbol{\beta}_P + \mathbf{Order}_{iq}\,\boldsymbol{\beta}_O + \mathbf{X}_i\,\boldsymbol{\beta}_X + \mathbf{u}_i + e_{iq}$, where i indexes participants and q denotes policy evaluations. Variables subscripted iq vary across observations within a participant (the experimental condition assigned to that policy, the order in which it was seen, and the response); Policy q is constant across participants seeing the same policy; $\mathbf{X}_i$ and $\mathbf{u}_i$ vary only across participants.

The outcome Y_{iq} refers to one of our dependent variables, measured on a 0–100 scale. The vector $\mathbf{D}_{iq}$ refers to the experimental manipulation with three conditions – predistribution, redistribution, and control – entered as a set of indicator variables with one omitted reference category. We include fixed effects $\mathbf{Policy}_q$ and $\mathbf{Order}_{iq}$ for the policy shown to the participant and its presentation order in the survey, each entered as indicator vectors with one omitted reference level. The vector $\mathbf{X}_i$ contains participant background characteristics measured pretreatment: perceived government efficacy, empathy, inequality perceptions, mobility perceptions, political ideology, party identification, education, income, age, gender, and race (categorical variables are coded with one reference category). For moderation analyses using party identification (see OSF), we code leaning independents as the party they lean toward (18). To account for repeated observations from the same participant, the model includes a participant-specific random intercept $\mathbf{u}_i \sim N(0, \sigma_u^2)$, which allows individuals to have different baseline levels of support. The remaining unexplained variation is captured by the idiosyncratic error $e_{iq} \sim N(0, \sigma^2)$. Full question wording for the variables used in these analyses is given in Sections A.9 and A.11.

A.8 Typographical errors in the preregistration document (Study 2)

The preregistration document contained a typographical omission in the predistributive frame for the Progressive Income Taxation policy, in which the words “more progressive” were inadvertently omitted, yielding the ungrammatical sentence “Making the income tax structure means people with low incomes take home more of their pay.” The fielded survey experiment included the grammatically complete sentence “Making the income tax structure more progressive means people with lower incomes take home more of their pay,” which paralleled the corresponding redistributive frame on the same page. The *SI Appendix* reproduces the policy descriptions exactly as they appeared to participants in the fielded survey experiment.

The preregistration document also contained a typographical error in the description of the Party ID variable. The text grouped “Closer to the Republican party,” “Neither,” and “Closer to the Democratic party” as Independents. The preregistered data preparation script, uploaded alongside the preregistration document to the OSF repository, instead classifies leaning independents with their preferred party. We follow the classification implemented in this preregistered script.

A.9 Pre-treatment questions (Study 2)

Background characteristics

- Age
  - Question: "What is your year of birth?"
  - Response format: Numeric text entry. Age computed as 2025 minus year of birth; missing values imputed with the sample median.

- Partisanship
  - Question: "Generally speaking, do you usually think of yourself as a Republican, a Democrat, an Independent, or what?"
  - Response options: Republican / Independent / Democrat / Other (please specify).
  - Follow-up if Republican: "Would you call yourself a strong Republican or a not very strong Republican?" (Strong Republican / Not very strong Republican)
  - Follow-up if Democrat: "Would you call yourself a strong Democrat or a not very strong Democrat?" (Strong Democrat / Not very strong Democrat)
  - Follow-up if Independent: "Do you think of yourself as closer to the Republican or Democratic Party?" (Closer to Republican party / Neither / Closer to Democratic Party)

- Political Ideology
  - Question: "Please consider the ENTIRE political spectrum. Do you usually think of yourself as conservative, moderate, or liberal?"
  - Scale: 1 ("Extremely Liberal") to 7 ("Extremely Conservative")

- Gender
    - Question: "What is your gender?"
    - Response options: Male / Female / Other.

- Race / Ethnicity
    - Question: "Please select which race / ethnicity you most identify as."
    - Response options: White / Caucasian; Black / African-American; Latino / Hispanic; Asian / Asian-American; Other.

- Education
    - Question: "What is the highest level of school that you have completed?"
    - Response options: Less than high school; High school diploma / GED; Some college or Associate's degree; Bachelor's degree; Postgraduate (Master's degree, Ph.D., Professional degree).

- Household Income
    - Question: "Approximately what is your current total household income before taxes? Please include all sources of income."
    - Response options: Less than $10,000; $10,000–19,999; $20,000–29,999; $30,000–39,999; $40,000–49,999; $50,000–59,999; $60,000–69,999; $70,000–79,999; $80,000–89,999; $90,000–99,999; $100,000–124,999; $125,000–174,999; $175,000–199,999; $200,000+.
    - Converted to a continuous variable using bracket midpoints, with the open-ended top category imputed via the method described in (2). Income was then rescaled to thousands of dollars.

Perceptions and attitudes

- Perceptions of Income Inequality
    - Question: "Please rate how much you agree or disagree with the following statements.
        - The gap between the incomes of the rich and the poor is too large these days.
        - Addressing income inequality should be a high priority for leaders in society."
    - Scale: 0 ("Strongly Disagree") to 100 ("Strongly Agree"). Two items averaged to form a composite.

- Perceptions of Social Mobility
    - Question: "Please rate how much you agree or disagree with the following statements.
        - People these days have much less of a chance to move up in society than they deserve.
        - Addressing social mobility should be a high priority for leaders in society."

    - Scale: 0 ("Strongly Disagree") to 100 ("Strongly Agree"). Two items averaged to form a composite.

- Perceived Government Efficacy
    - Question: "Please rate how much you agree or disagree with the following statements.
        - Our government can be a useful tool for fixing social problems in the country.
        - Government action is an effective way to address social problems in the United States."
    - Scale: 0 ("Strongly Disagree") to 100 ("Strongly Agree"). Two items averaged to form a composite.

- Empathy for the Poor
    - Question: "Please rate how much you agree or disagree with the following statements.
        - Others' economic misfortunes do not disturb me that much. [reverse-coded]
        - I feel great concern for Americans born in poverty.
        - I don't feel very sorry for poor people. [reverse-coded]
        - I feel a great deal of empathy for poor Americans."
    - Scale: 0 ("Strongly Disagree") to 100 ("Strongly Agree"). Four items, with items 1 and 3 reverse-coded, averaged to form a composite.

A.10 Experimental manipulation of policy descriptions text (Study 2)

Below, we document the text describing the policies in the three experimental conditions. The text formatted in bold face reflects how the text was presented to study participants in the experiment. Each policy is numbered P1–P6, and each of its three descriptions is tagged with the policy number plus a condition letter: c (control), p (predistributive), and r (redistributive). These codes index the descriptions in the Policy Types and Policy Descriptions section in A.3.

1. *Earned Income Tax Credit (EITC)*

Control (P1c):
'**Increase the Earned Income Tax Credit.**

The Earned Income Tax Credit (EITC) is a federal income tax credit available to employed individuals who earn low or middle incomes. For employed individuals who earn below the policy's income threshold, the EITC offers a tax credit, supplementing their take-home pay.'

Predistributive Description (P1p):
'**Increase the Earned Income Tax Credit.**

The Earned Income Tax Credit (EITC) is a federal income tax credit available to employed individuals who earn low or middle incomes. For employed individuals who earn below the policy's income threshold, the EITC offers a tax credit, supplementing their take-home pay.

Increasing the EITC would **increase the take-home pay for employed people who work for low or middle incomes**.'

Redistributive Description (P1r):
'**Increase the Earned Income Tax Credit.**

The Earned Income Tax Credit (EITC) is a federal income tax credit available to employed individuals who earn low or middle incomes. For employed individuals who earn below the policy's income threshold, the EITC offers a tax credit, supplementing their take-home pay.

Increasing the EITC would **redistribute money in society from high-income people** - who pay more in taxes - to lower-income people - who are eligible to receive the tax credit.'

2. *Increase in the Federal Minimum Wage*

Control (P2c):
'**Increase the Federal Minimum Wage to $12 an hour.**

The Federal Minimum Wage is a legally-mandated wage floor, the minimum hourly rate employers in the U.S. are required to pay employees. It is currently $7.25.'

Predistributive Description (P2p):
'**Increase the Federal Minimum Wage to $12 an hour.**

The Federal Minimum Wage is a legally-mandated wage floor, the minimum hourly rate employers in the U.S. are required to pay employees. It is currently $7.25.

Raising the minimum wage to $12 an hour would **ensure many low-wage workers earn more for their work**.'

Redistributive Description (P2r):
'**Increase the Federal Minimum Wage to $12 an hour.**

The Federal Minimum Wage is a legally-mandated wage floor, the minimum hourly rate employers in the U.S. are required to pay employees. It is currently $7.25.

Raising the minimum wage to $12 an hour would **redistribute resources from employers to low-wage workers**.'

3. *Progressive Income Taxation*

Control (P3c):
'**Make the income tax system more progressive.**

In the U.S., people with higher incomes pay a higher income tax rate than those with lower incomes. Making the tax system more progressive means further increasing the tax rates for those with high incomes and decreasing them for those with lower incomes.'

Predistributive Description (P3p):
'**Make the income tax system more progressive.**

In the U.S., people with higher incomes pay a higher income tax rate than those with lower incomes. Making the tax system more progressive means further increasing the tax rates for those with high incomes and decreasing them for those with lower incomes.

Making the income tax structure more progressive means people with lower incomes **take home more of their pay**.'

Redistributive Description (P3r):
'**Make the income tax system more progressive.**

In the U.S., people with higher incomes pay a higher income tax rate than those with lower incomes. Making the tax system more progressive means further increasing the tax rates for those with high incomes and decreasing them for those with lower incomes.

Making the income tax structure more progressive means that **a larger share of taxes is collected from high-income earners** than those with lower incomes.'

4. *Protecting the Right to Organize Act (PRO Act)*

Control (P4c):
'**Strengthen labor unions and collective bargaining.**

The Protecting the Right to Organize Act (PRO Act) is a proposed U.S. law that would make it easier for workers to form and join unions. The policy clarifies rules for union elections, sets guidelines for employer conduct, and reduces barriers to organizing.'

Predistributive Description (P4p):
'**Strengthen labor unions and collective bargaining.**

The Protecting the Right to Organize Act (PRO Act) is a proposed U.S. law that would make it easier for workers to form and join unions. The policy clarifies rules for union elections, sets guidelines for employer conduct, and reduces barriers to organizing.

Stronger unions can more effectively negotiate higher wages. As a result, **workers get higher wages for their work**.'

Redistributive Description (P4r):
'**Strengthen labor unions and collective bargaining.**

The Protecting the Right to Organize Act (PRO Act) is a proposed U.S. law that would make it easier for workers to form and join unions. The policy clarifies rules for union elections, sets guidelines for employer conduct, and reduces barriers to organizing.

Stronger unions can more effectively negotiate higher wages. As a result, some of the company's **revenue that would have gone to owners and shareholders is instead redistributed to employees**.'

5. *Medicare For All Who Want It*

Control (P5c):
‘**Make healthcare more affordable and accessible.**

“Medicare For All Who Want It” is a policy that provides government-run healthcare insurance. It aims to make healthcare more affordable and accessible. People can choose the public plan if they prefer, but private insurance is still available for those who want to keep it.’

Predistributive Description (P5p):
‘**Make healthcare more affordable and accessible.**

“Medicare For All Who Want It” is a policy that provides government-run healthcare insurance. It aims to make healthcare more affordable and accessible. People can choose the public plan if they prefer, but private insurance is still available for those who want to keep it.

The policy **helps workers lower their health insurance costs**. By reducing the financial burden of healthcare, the policy allows workers to **keep more of their earnings**.’

Redistributive Description (P5r):
‘**Make healthcare more affordable and accessible.**

“Medicare For All Who Want It” is a policy that provides government-run healthcare insurance. It aims to make healthcare more affordable and accessible. People can choose the public plan if they prefer, but private insurance is still available for those who want to keep it.

The policy is financed in a way that **asks higher-income earners to contribute more while those with lower incomes pay less**. This arrangement **redistributes healthcare costs**, shifting financial responsibility away from those with fewer resources onto those with more.’

6. *America's College Promise Act*

Control (P6c):
'**Make community college education free.**

The America's College Promise Act is a policy that aims to provide free community college education for students from a low-income background.'

Predistributive Description (P6p):
'**Make community college education free.**

The America's College Promise Act is a policy that aims to provide free community college education for students from a low-income background.

Making community college education free helps students from low-income backgrounds gain practical skills so that they can **achieve higher incomes through their work**.'

Redistributive Description (P6r):
'**Make community college education free**.

The America's College Promise Act is a policy that aims to provide free community college education for students from a low-income background.

Making community college education free helps students from low-income backgrounds gain practical skills, **while the costs are covered by taxes mostly from those with higher incomes**.'

A.11 Post-treatment questions (Study 2)

We use the following post-treatment questions in Study 2. Respondents answered questions after each policy. When multiple items are listed, we averaged them to form the preregistered composite.

Main dependent variable

- Policy Support
  - Question: "Please rate how much you agree or disagree with the following statements.
    - I support this policy.
    - The effects of this policy would generally be good."
  - Scale: 0 ("Strongly Disagree") to 100 ("Strongly Agree"). Two items averaged to form a composite.

Secondary dependent variable

- Candidate Support
  - Question: "Please rate how much you agree or disagree with the following statements.
    - If a politician supported this policy, I would be more likely to vote for them.
    - I would view a politician more favorably if they supported this policy."
  - Scale: 0 ("Strongly Disagree") to 100 ("Strongly Agree"). Two items averaged to form a composite.

Policy perceptions

- Respect for Hard Work
  - Question: "Please rate how much you agree or disagree with the following statements.
    - This policy would ensure that hard work pays off.
    - This policy would pay respect to hard-working Americans."
  - Scale: 0 ("Strongly Disagree") to 100 ("Strongly Agree"). Two items averaged to form a composite.

- Societal Fairness
  - Question: "Please rate how much you agree or disagree with the following statements.
    - This policy would make society more fair.
    - This policy would help ensure that everyone has a fair chance to achieve what they want."
  - Scale: 0 ("Strongly Disagree") to 100 ("Strongly Agree"). Two items averaged to form a composite.

- Beneficiaries Would Not Burden Taxpayers

- Question: "Please rate how much you agree or disagree with the following statements.
  - Those who would benefit from this policy place an unfair strain on taxpayers." [reverse-coded]
- Scale: 0 ("Strongly Disagree") to 100 ("Strongly Agree")

- Beneficiary Deservingness
  - Question: "Please rate how much you agree or disagree with the following statements.
    - Those who would benefit from this policy deserve the benefit they receive."
  - Scale: 0 ("Strongly Disagree") to 100 ("Strongly Agree")

- Benefits to Society
  - Question: "Please rate how much you agree or disagree with the following statement.
    - Overall, our society would be better off if this policy was implemented."
  - Scale: 0 ("Strongly Disagree") to 100 ("Strongly Agree")

- Personal Benefits
  - Question: "Please rate how much you agree or disagree with the following statement.
    - I would be better off if this policy was implemented."
  - Scale: 0 ("Strongly Disagree") to 100 ("Strongly Agree")

- Gov't Could Effectively Implement
  - Question: "Please rate how much you agree or disagree with the following statement.
    - The government would effectively implement this policy."
  - Scale: 0 ("Strongly Disagree") to 100 ("Strongly Agree")

- Minorities Would Primarily Benefit
  - Question: "Please rate how much you agree or disagree with the following statement.
    - This policy would primarily benefit racial and ethnic minorities in the U.S."
  - Scale: 0 ("Strongly Disagree") to 100 ("Strongly Agree")

Manipulation checks

The below manipulation check questions were shown only for the first three policies each participant saw, after the main outcome and mechanism questions. Their condition effects are therefore estimated only on that subsample. Note that policy order was randomized.

We form a perceived-type composite, our main text measure of perceived type, by averaging the predistribution item with the reverse-coded "redistribution from and to" item, so that higher values indicate a policy is perceived as more predistributive than redistributive .

- Predistribution
    - Question: "Please rate how much you agree or disagree with the following statements.
        - This policy aims to increase the wages of low-income workers."
    - Scale: 0 ("Strongly Disagree") to 100 ("Strongly Agree")

- Redistribution from and to
    - Question: "Please rate how much you agree or disagree with the following statements.
        - This policy aims to reduce economic inequality by taking resources from the rich and giving resources to the poor."
    - Scale: 0 ("Strongly Disagree") to 100 ("Strongly Agree")

- Redistribution from
    - Question: "Please rate how much you agree or disagree with the following statements.
        - This policy aims to reduce economic inequality by taking resources from the rich."
    - Scale: 0 ("Strongly Disagree") to 100 ("Strongly Agree")

- Redistribution to
    - Question: "Please rate how much you agree or disagree with the following statements.
        - This policy aims to reduce economic inequality by giving resources to people with lower incomes."
    - Scale: 0 ("Strongly Disagree") to 100 ("Strongly Agree")

SI References

1. J. Mummolo, E. Peterson, Improving the Interpretation of Fixed Effects Regression Results. Political Science Research and Methods 6, 829–835 (2018).
2. M. Hout, "Getting the Most Out of the GSS Income Measures" (University of California, 2004).
3. American National Election Studies, ANES 2016 Time Series Study Full Release. Dataset and documentation. September 4, 2019 version. Stanford University and University of Michigan (2019).
4. American National Election Studies, ANES 2020 Time Series Study Full Release. Dataset and documentation. February 10, 2022 version (2021).
5. S. Ansolabehere, B. Schaffner, CCES Common Content, 2015. Harvard Dataverse (2017). https://doi.org/10.7910/DVN/SWMWX8.
6. S. Ansolabehere, B. Schaffner, CCES Common Content, 2016. Harvard Dataverse (2017). https://doi.org/10.7910/DVN/GDF6Z0.
7. B. Schaffner, S. Ansolabehere, CCES Common Content, 2017. Harvard Dataverse (2019). https://doi.org/10.7910/DVN/3STEZY.
8. B. Schaffner, S. Ansolabehere, S. Luks, CCES Common Content, 2018. Harvard Dataverse (2019). https://doi.org/10.7910/DVN/ZSBZ7K.
9. B. Schaffner, S. Ansolabehere, S. Luks, CCES Common Content, 2019. Harvard Dataverse (2020). https://doi.org/10.7910/DVN/WOT7O8.
10. B. Schaffner, S. Ansolabehere, S. Luks, Cooperative Election Study Common Content, 2020. Version 4. Harvard Dataverse (2021). https://doi.org/10.7910/DVN/E9N6PH.
11. B. Schaffner, S. Ansolabehere, CES Common Content, 2021. Harvard Dataverse (2022). https://doi.org/10.7910/DVN/OPQOCU.
12. B. Schaffner, S. Ansolabehere, M. Shih, Cooperative Election Study Common Content, 2022. Version 4. Harvard Dataverse (2023). https://doi.org/10.7910/DVN/PR4L8P.
13. M. Davern, R. Bautista, J. Freese, P. Herd, S. L. Morgan, General Social Survey 1972–2024 (NORC edition; 2024 Release 1). 1 datafile and 1 codebook. Chicago: NORC at the University of Chicago (2025).
14. Gallup, Inc., Gallup Poll Social Series. Version 14.0. Redivis. (2025). https://doi.org/10.57761/vxfa-he67.
15. Understanding America Study, Understanding America Study (UAS): Waves 167, 199, 280, 296, and 322. USC Dornsife Center for Economic and Social Research, University of Southern California. Waves fielded 2019–2023 (2023). https://uasdata.usc.edu/.
16. Heldrich Center for Workforce Development, Topline Survey Results: Work Trends Pre-election and Post-election Surveys. Published May 18, 2021. Edward J. Bloustein School of Planning and Public Policy, Rutgers University. Pre-election survey: Oct 21–Nov 1, 2020 (N=810). Post-election survey: Dec 4–14, 2020 (N=814). Web-based surveys conducted by Ipsos Public Affairs (2021).
17. Heldrich Center for Workforce Development, Work Trends April 2023 Survey. Survey fielded April 7–10, 2023 on the SSRS Opinion Panel Omnibus platform, N=1,026 U.S. adults. Related report: C. E. Van Horn and J. Starace, Whipsawed, Precarious, and Pessimistic: Americans' Changing Views About the Economy and Labor Market During a Decade of Fundamental Change (May 2023). Edward J. Bloustein School of Planning and Public Policy, Rutgers University (2023).
18. J. R. Petrocik, Measuring party support: Leaners are not independents. *Electoral Studies* **28**, 562–572 (2009).
